# Case Report: Structural Changes in the Coronary Vessel Wall in a Patient With Incomplete Kawasaki Disease

**DOI:** 10.3389/fped.2022.845723

**Published:** 2022-03-03

**Authors:** Takamichi Ishikawa, Hiroki Uchiyama, Satoshi Mogi, Hayato Ohtani

**Affiliations:** ^1^Department of Pediatrics, Hamamatsu University School of Medicine, Hamamatsu, Japan; ^2^Division of Cardiology, Internal Medicine 3, Hamamatsu University School of Medicine, Hamamatsu, Japan

**Keywords:** Kawasaki disease, incomplete Kawasaki disease, optical coherence tomography, coronary arterial lesion, intravenous immunoglobulin

## Abstract

**Background:**

Kawasaki disease (KD) is an acute systemic vasculitis of infants and young children that affects medium-sized vessels. Conventional cardiac imaging techniques, such as cardiac catheterization, are useful for characterizing the coronary arterial lesion (CAL) size and luminal diameter of the diseased coronary artery segment in patients with KD, but there are limitations to the visualization of the detailed vascular anatomy. Optical coherence tomography (OCT) is a high-resolution intracoronary arterial imaging modality that can distinguish the three layers of the coronary arterial wall. Several studies have reported coronary artery wall abnormalities in KD patients with coronary arterial aneurysm or regressed aneurysm. However, there have been no reports on changes in the coronary artery wall in cases of incomplete KD without CAL.

**Case Presentation:**

We herein report an 11-year-old girl with a history of incomplete KD without coronary arterial aneurysms. She had been diagnosed with perimembranous ventricular septal defect (VSD) after birth and had experienced incomplete KD at 1 year old. During her hospitalization for KD, she did not receive intravenous immunoglobulin (IVIG), because she did not meet the Harada score or criteria for treatment in patients with incomplete KD established by the American Heart Association. No dilatation or coronary artery aneurysm were observed on transthoracic echocardiography in the acute or follow-up period. At 11 years old, she received cardiac catheterization and coronary angiography (CAG) for the evaluation of a VSD and follow-up of KD. CAG demonstrated no aneurysm, dilatation, or significant stenosis of the coronary arteries. We performed an OCT study, which revealed the presence of intimal thickening, disruption of the media, and neovascularization in the left anterior descending artery.

**Conclusion:**

OCT demonstrates the structural changes of CA even in the patient with incomplete KD who have not been treated with IVIG.

## Introduction

Kawasaki disease (KD) is an acute systemic vasculitis of infants and young children that affects medium-sized vessels. Coronary arterial lesions (CALs) are a serious complication occurring in 5% of treated and 25–30% of untreated KD patients (1, 2). Conventional cardiac imaging techniques, such as echocardiography, computed tomography, coronary angiography, cardiac magnetic resonance imaging, and invasive angiography, are useful for determining the aneurysm size and luminal diameter of the diseased coronary artery segment. However, those modalities are of limited use for the visualization of detailed vascular anatomical data.

Optical coherence tomography (OCT) is a high-resolution intracoronary arterial imaging modality that is able to distinguish the three layers of the coronary arterial wall (3). However, while several studies have used OCT to evaluate CAL in patients with KD (4–6), there have been no reports of changes in the coronary artery wall in cases of incomplete KD without CAL.

## Case Description

An 11-year-old Japanese girl was admitted to undergo cardiac catheterization and coronary angiography (CAG) for the evaluation of a ventricular septal defect (VSD) and follow-up of KD.

### Past Illness

A heart murmur had been detected after birth, and she had been diagnosed with perimembranous VSD by echocardiography. She had been followed-up for VSD at the outpatient unit periodically.

At 17 months old, she was referred by an affiliated clinic for a fever for the past 2 days, polymorphous rash, and erythema of the hand. On admission, laboratory test findings were as follows: white blood cell counts 8,000/mm^3^, hemoglobin 14.4 g/dL, hematocrit 41.0%, platelet count 20.3 × 10^4^/mm^3^, serum albumin 4.4 g/dL, alanine transaminase 38 IU/L, and C-reactive protein (CRP) 8.59 mg/dL. Blood culture and serology of Epstein Barr virus to rule out the infectious origin of symptoms were negative. She was diagnosed with incomplete KD. Oral treatment with medium-dose acetylsalicylic acid (30 mg/kg/day) immediately improved these symptoms. Four days after admission, the CRP level decreased to 1.25 mg/dL. She was not on intravenous immunoglobulin (IVIG), because her Harada score was <4/7 (2/7) (7), and she did not meet the criteria for incomplete KD treatment established by the American Heart Association (1).

**Figure 1:**
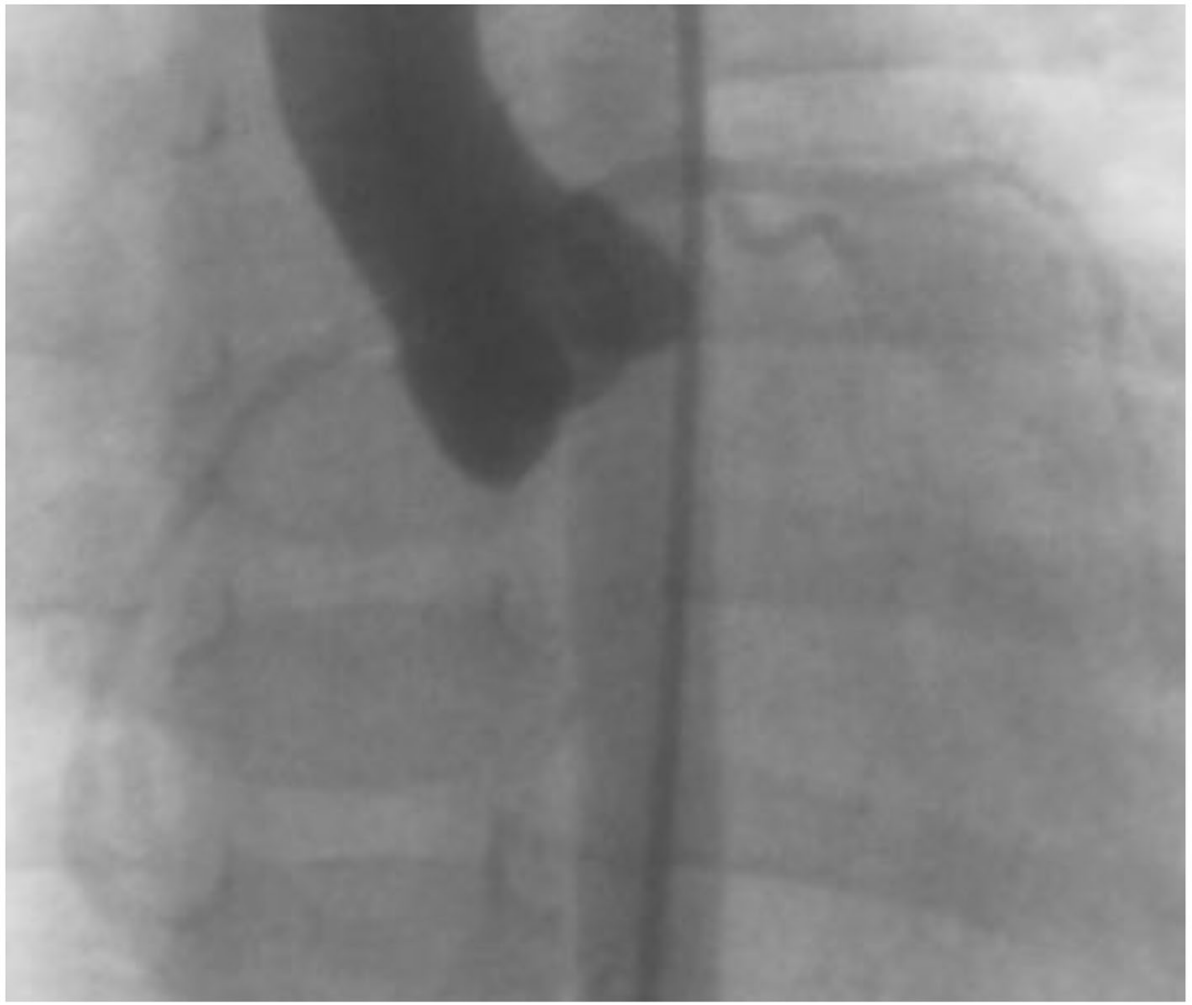
An aortic angiogram showing no coronary artery aneurysm.

After the fever had disappeared, low-dose aspirin therapy (5 mg/kg/day) was started and then completed 2 months later. No dilatation or coronary artery aneurysm was observed on transthoracic echocardiography in the acute or follow-up period. There were no obvious coronary aneurysms on the cardiac catheterization to evaluate the VSD 3 months after the onset of KD (Figure 1). She was followed up for VSD without surgery, as her pulmonary blood flow/systemic blood flow ratio (Qp/Qs) was 1.3.

### Findings on Admission and Management

Her height and weight were 139.9 cm (−0.5 standard deviations [SD]) and 33.8 kg (−0.4 SD), respectively. Her heart rate was 80 bpm, and blood pressure was 96/60 mmHg. A physical examination revealed a grade 4/6 pansystolic murmur at the left sternal border in the third to the fourth intercostal space. The liver was not palpable below the right costal margin. There were no abnormal findings in the abdomen or extremities or irregular and blood chemistry test results.

The cardiothoracic ratio on chest X-ray was 52%. The 12-lead electrocardiogram (ECG) revealed mild left ventricular hypertrophy and normal sinus rhythm without ST-T segment change or an abnormal Q wave. Exercise stress testing showed no abnormal findings on ECG and the patient had no symptoms at maximum effort. Two-dimensional transthoracic echocardiography showed a 5.0-mm perimembranous VSD with left-to-right shunt and a normal left ventricular ejection fraction of 70% without local asynergy.

**Figure 2:**
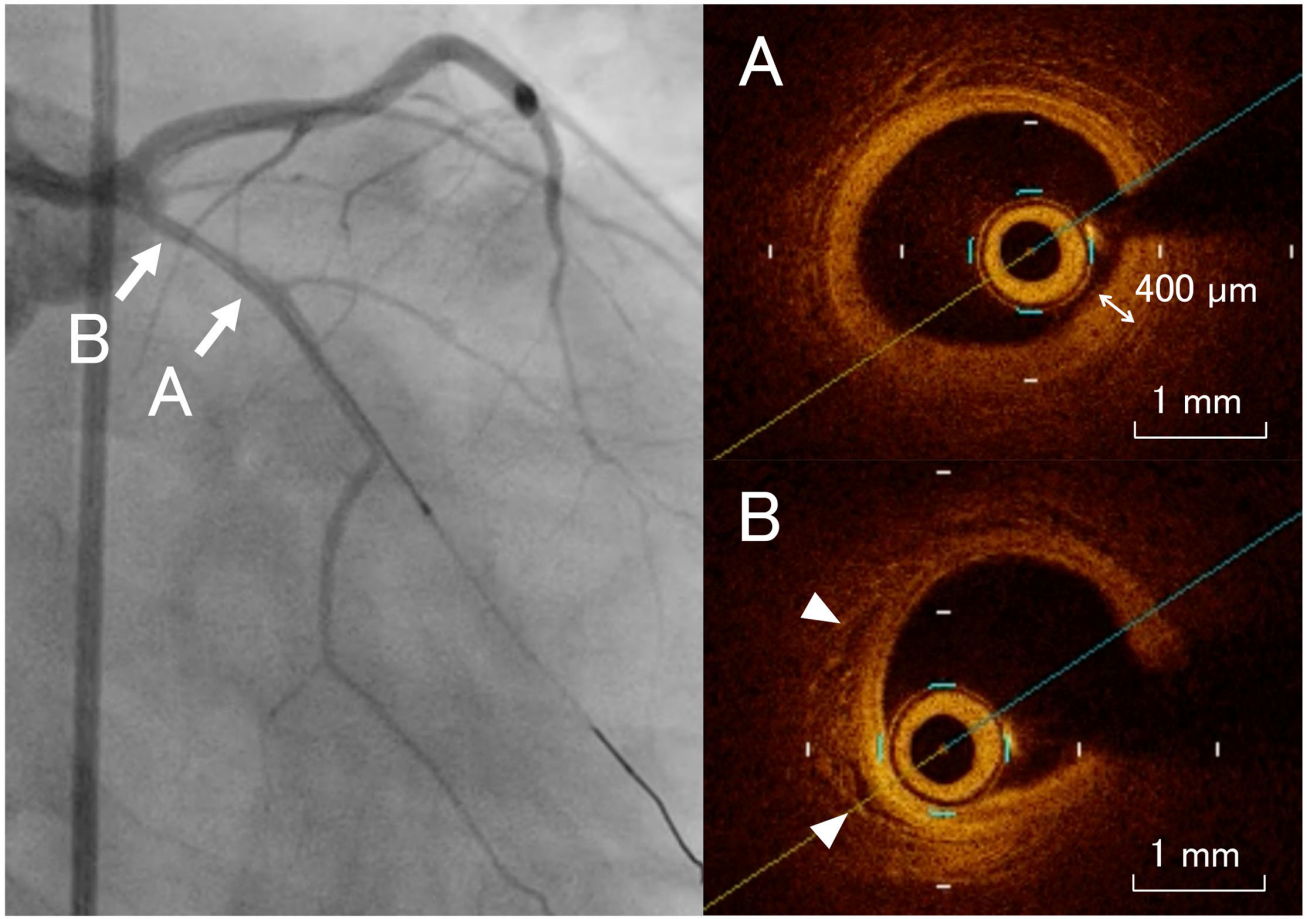
Coronary angiogram images with the corresponding optical coherence tomography examinations. The left anterior descending artery shows intimal thickening, disruption of the media **(A)**, and neovascularization [**(B)** arrowheads].

CAG demonstrated no aneurysm, dilatation, or significant stenosis of the coronary arteries. We performed an OCT study (ILUMIEN™ OCT Imaging System: St. Jude/LightLab, St. Paul, MN, USA), which revealed the presence of intimal thickening, disruption of the media, and neovascularization in the left anterior descending artery (Figure 2). There were no abnormal OCT findings in the other coronary artery branches.

CAG and OCT were approved by the Ethics Committee of Hamamatsu University School of Medicine (18-029), and the parents provided their informed consent. The patient received surgical closure of the VSD, as the Qp/Qs ratio was 1.5 on cardiac catheterization. No coronary artery abnormalities were observed in the perioperative period.

## Discussion

This patient with incomplete KD had intimal thickening, disruption of the media, and neovascularization in the coronary artery detected by OCT. KD is characterized by severe systemic vasculitis and an acute febrile illness in children. This inflammatory process has been shown to persist for many years after the initial diagnosis, with the proliferation of dense smooth muscle cells and accumulation of fibrous tissue, leading to intimal thickening (8). Progressive intimal hyperplasia is associated with hypoxia of the cells, which further promotes angiogenesis through hypoxia-inducible transcription factors that then induce the transcription of proangiogenic genes (9). These findings suggest the importance of the precise evaluation of coronary wall structural changes in at-risk KD patients.

OCT is a high-resolution intracoronary arterial imaging modality that is able to distinguish the three layers of the coronary arterial wall: the intima, media, and adventitia (3). Several studies have used OCT to evaluate CAL in patients with KD (4–6). A previous study reported that calcification and destruction of the media layer, neovascularization, and white thrombi were found in not only segments with aneurysm, but also in those with regressed aneurysm (6). However, there have been no previous reports of changes in the coronary artery wall in cases of incomplete KD without CAL.

The present patient did not receive IVIG therapy, as she did not meet the Harada score cut-off (7) or the criteria for the treatment of patients with incomplete KD established by the American Heart Association (1). In cases with incomplete KD, the timely commencement of IVIG therapy is often hampered by the lack of definitive diagnostic symptoms. As a result, some incomplete KD patients experience spontaneous defervescence before IVIG administration as in the present case. However, it has been suggested that there is persistent mild systemic inflammation in incomplete KD patients who experience spontaneous defervescence without receiving IVIG (10). Patients with KD who are not treated with IVIG therapy have a 25–30% risk of developing CALs (1, 2). Other studies have shown that the CAL complication rate within 30 days of illness in the KD patients received aspirin alone was 20–40% (1, 11). In the previous reports, all KD patients with abnormal findings on OCT were diagnosed with complete KD and/or received delayed administration of IVIG or showed IVIG resistance (4–6). Prompt IVIG therapy is the currently internationally recognized method of managing KD with the specific aim of preventing CALs (1). However, these findings suggest that it is possible to prevent not only CALs but also structural changes of the coronary vessel wall if IVIG is administrated without delay.

The present case had abnormalities of the coronary artery wall, despite a lack of CALs. While the cause and pathogenesis of these findings were unclear, previous studies have shown that intimal thickening, vascular endothelial dysfunction, and impaired coronary microcirculation are observed, even in patients with apparently normal coronary arteries (12, 13). Coronary artery remodeling and neoangiogenesis may continue for a decade after the development of acute KD (8). Previous studies of coronary artery specimens from KD patients have demonstrated the phenomenon of vascular senescence, which is similar to atherosclerosis in adults (14, 15). The use of OCT to identify atherosclerotic plaque and detect calcification in adults with coronary artery disease has been well described. The correlations between the pathological and OCT findings have described in this population (16). Because this procedure was introduced in this field very recently, the number of reports that have investigated the coronary arteries of KD patients by OCT has been limited. However, a recent report using OCT demonstrated that after KD 5–40% of patients had intimal hyperplasia, fibrosis, and cellular infiltration in segments with no history of aneurysm or coronary dilatation (6). These findings suggest that OCT can detect structural abnormalities in the coronary vessel wall that are missed by conventional imaging.

The findings in the present and past reports suggest that we should be aware of not only CALs but also structural changes of the coronary vessel wall when we encounter patients who have not been treated with IVIG or for whom the administration of IVIG is delayed or patients who show IVIG resistance. Vascular structural abnormalities lead to vascular dysfunction and impaired blood flow. Endothelial dysfunction, which is caused by endothelial damage, is an early feature of atherosclerosis (17, 18). Therefore, we believe that these patients may require careful follow-up with monitoring to detect the future development of arteriosclerosis. In addition, the prevention of additional cardiovascular risk factors, such as obesity, hypertension, hyperlipidemia, and smoking may be required in such patients. Further large-scale and prospective studies to evaluate the vascular structure and the association with an increased risk of late adverse events in these patients are needed. The accumulation of evidence using OCT may contribute to risk stratification and the construction of a management system for KD patients.

## Data Availability Statement

The raw data supporting the conclusions of this article will be made available by the authors, without undue reservation.

## Ethics Statement

The studies involving human participants were reviewed and approved by the Ethics Committee of Hamamatsu University School of Medicine. Written informed consent to participate in this study was provided by the participants' legal guardian/next of kin.

## Author Contributions

TI wrote the manuscript. All authors listed have made a substantial, direct, and intellectual contribution to the work and approved it for publication. All authors contributed to the article and approved the submitted version.

## Funding

This work was supported by JSPS KAKENHI Grant Number JP18K07787.

## Conflict of Interest

The authors declare that the research was conducted in the absence of any commercial or financial relationships that could be construed as a potential conflict of interest.

## Publisher's Note

All claims expressed in this article are solely those of the authors and do not necessarily represent those of their affiliated organizations, or those of the publisher, the editors and the reviewers. Any product that may be evaluated in this article, or claim that may be made by its manufacturer, is not guaranteed or endorsed by the publisher.
